# Enteric involvement of SARS-CoV-2: Implications for the COVID-19 management, transmission, and infection control

**DOI:** 10.1080/21505594.2020.1794410

**Published:** 2020-07-27

**Authors:** Jiandong Shi, Jing Sun, Yunzhang Hu

**Affiliations:** Institute of Medical Biology, Chinese Academy of Medical Sciences and Peking Union Medical College, Kunming, Yunnan, China; Yunnan Provincial Key Laboratory of Vector-borne Diseases Control and Research, Pu’er, Yunnan, China

**Keywords:** COVID-19, SARS-CoV-2, fecal-oral transmission, mode of transmission, digestive tract route

A novel viral pneumonia referred to as coronavirus disease 2019 (COVID-19), has been rapidly spread worldwide since its first outbreak in Wuhan, China, in December 2019 [1]. The disease has caused a global pandemic that has affected over 213 countries and territories and poses a severe threat to public health [2]. As of 7 June 2020, the World Health Organization confirmed 6,799,713 COVID-19 cases worldwide, including 397,388 (5.84%) deaths [3]. COVID-19 is the third introduction of a deadly coronavirus that spreads from wildlife into human populations. This infection has not only been affecting the medical system, but also the global economy, and has even completely changed people’s lives.

The most usual symptoms of COVID-19 are fever, cough, sore throat, shortness of breath, headache, fatigue, and muscle ache. The clinical course of COVID-19 is characterized by a wide range of severity and progression patterns. Mild symptoms are manifested in the vast majority of people. However, in the elderly and patients with complications, it may progress to pneumonia, acute respiratory distress syndrome (ARDS), and severe sepsis with shock and multiple organ dysfunction [4,5]. In a previous study, the incubation period of the virus was found to be between 2 and 14 days, with an average length of 5.2 days [6]. The mortality rate of adult inpatients ranged from 4% to 11%, and the total case fatality rate was between 2% and 3% [7]. Currently, no effective drugs and vaccines exist for the prevention and treatment of COVID-19. Severe acute respiratory syndrome coronavirus 2 (SARS-CoV-2), a new type of β-coronavirus, is responsible for the new COVID-19 acute respiratory disease. It is generally believed that the virus has originated in bats and has then spread to humans through unknown intermediate animals. SARS-CoV-2 spreads from person to person mainly through the transmission of virus droplets or aerosols directly from the respiratory tract of infectious individuals to the mucosal surfaces of recipients via eyes, mouth, or nose [8]. Thus, for an emerging virus, it is of great significance for the prevention and control of the virus to fully elucidate its transmission route and infection mode. However, other routes and modes of transmission, such as fecal-oral, are yet to be elucidated.

In addition to fever and respiratory symptoms, gastrointestinal (GI) symptoms, such as anorexia, diarrhea, vomiting, nausea, abdominal pain, and gastrointestinal bleeding, are common in COVID-19 patients [9–11]. The recent data from Wuhan showed that up to 79% of the patients had gastrointestinal symptoms, such as diarrhea, decreased appetite, nausea, vomiting, abdominal pain, and gastrointestinal bleeding during the onset and subsequent hospitalization [12]. A study summarizing data of 2,023 COVID-19 patients revealed the percentage of various gastrointestinal symptoms, including anorexia (39.9%-50.2%) in adults, diarrhea (2%-49.5%) both in adults and children, vomiting (3.6%-15.9% of adult patients and 6.5%-66.7% in children), nausea (1%-29.4%), abdominal pain (2.2%-6.0%), and gastrointestinal bleeding (4%-13.7%) in severely ill patients [13]. Diarrhea was the most common gastrointestinal symptom in children and adults, and vomiting was prominent in children. Abdominal pain was frequently observed in severely ill patients [13]. Notably, among COVID-19 patients with GI symptoms, as the severity of the disease increased, the GI symptoms were pronounced. Moreover, the patients without any symptoms of the GI tract have a higher cure rate than those with symptoms of the digestive tract (60% *vs*. 34.3%) [11]. This phenomenon could be attributed to either viral replication in the gastrointestinal tract causing severe disease. A recent study reported that 74 (11.4%) of 651 COVID-19 patients outside Wuhan had at least one Gl symptom (nausea, vomiting, or diarrhea) [14]. Importantly, the latest and most comprehensive meta-analysis, including 59,254 patients from 11 countries, revealed that 9% of the patients presented gastrointestinal symptoms [15]. Therefore, GI symptoms are a common complaint in patients with COVID-19 in addition to fever and/or respiratory symptoms at the time of hospital visit. The GI tract may be a potential transmission route and a target of SARS-Cov-2. Based on the existing clues, the GI symptoms of COVID-19 can be speculated to have been caused by the direct damage of the virus to the intestine, rather than by the immunopathogenic response caused by the host lung infection. So far, the infection control and surveillance of SARS-CoV-2 have mainly been focused on the respiratory system. The ignorance of the digestive system symptoms caused by SARS-CoV-2 may hamper the effective disease control. Therefore, clinicians worldwide, especially gastroenterologists, should pay more attention to patients with GI symptoms and new virus infection manifestations as they may require changes in the treatment strategy.

Angiotensin-converting enzyme 2 (ACE-2), the receptor of SARS-CoV-2, was found to be highly expressed in the small intestine, especially in proximal and distal enterocytes [16]. A study by Hashimoto et al. [17] reported the pathological importance of ACE2 in modulating intestinal inflammation and diarrhea, which suggested that ACE2-expressing small intestinal epithelium cells might be vulnerable to SARS-CoV-2. Another study by Xiao et al. confirmed that the intestinal tropism of SARS-CoV-2 could be one of the mechanisms of the gastrointestinal manifestations [18]. Xiao et al. showed that viral RNA and nucleocapsid protein were distributed in gastric, duodenal, and rectal epithelia in the patients [18]. The results demonstrated that human intestinal cells are highly susceptible to SARS-CoV-2 and support strong replication of the virus, suggesting that the human gastrointestinal tract is an alternative route of infection for SARS-CoV-2. Thus, gastrointestinal tropism might explain the frequent occurrence of diarrhea in SARS-CoV-2 infection. Another study by Liang et al. [19] also reported similar results that ACE2 was highly expressed in the human small intestine, especially in proximal and distal enterocytes. Thus, it could be deduced that the gastrointestinal tract is a target organ of SARS-CoV-2 infection and replication. However, the exact molecular mechanism of COVID-19, causing digestive symptoms, needs to be further investigated.

Current evidence shows that live virus particles of SARS-CoV-2 are excreted from feces in a subset of patients. In a recent study, Chen et al. reported 28 patients who tested positive for SARS-CoV-2 RNA in stool specimens [20]. Zhang et al. isolated viable SARS-CoV-2 from a stool sample of a COVID-19 patient about 15 days after the onset of the disease [21]. Wang et al. isolated live virus particles of SARS-CoV-2 from four SARS-CoV-2-positive fecal specimens; of these, two specimens were observed by electron microscopy [22]. Viral shedding in stools was also reported in asymptomatic patients. Tang et al. reported an asymptomatic child who was positive for SARS-CoV-2, as assessed by viral RNA test in a stool specimen 17 days after the last virus exposure [23]. Therefore, positive stool samples of asymptomatic COVID-19 patients suggest a new screening index for asymptomatic patients. Nonetheless, additional studies are required to determine if stool samples could be utilized as a screening tool for asymptomatic COVID-19 patients. Earlier, Xiao et al. detected viral RNA in the feces of 71 patients diagnosed with COVID-19. The authors found that 53.4% of the patients had viral RNA in the feces, whereas 23% of the patients had virus-positive stool, but their respiratory specimens were virus-negative [18]. These findings suggest that the gastrointestinal infection cause by the virus can persist for a long time, even after the removal of the respiratory virus. Therefore, for better monitoring and disease control, nucleic acids testing for SARS-CoV-2 of the feces of COVID-19 patients should be routinely performed. These findings highlight the urgency for further research on potential fecal-oral transmission and pathological significance of viral gastrointestinal infections. Thus, more information would be assimilated about how the coronavirus survives in the gut, how it causes gastrointestinal symptoms, such as diarrhea, how the virus survives in the stools at different temperatures, and whether the virus isolated from feces is still infectious. The potential role of fecal viral shedding on the virus pandemic spread and the disease development and outcomes in COVID-19 patients with gastrointestinal symptoms remains unclear. To elucidate the gastrointestinal involvement of SARS-CoV-2 infection, more research needs to be conducted in the future.

The presence of a live virus in stool samples is a new phenomenon for SARS-CoV-2, suggesting its new modes of transmission, such as the possibility of “fecal-oral” and “fecal-respiratory.” Coronavirus can cause respiratory and intestinal infections in humans and animals, leading to diseases with different clinical symptoms from common cold to severe pulmonary infection [24]. Two important members of the coronavirus family, severe acute respiratory syndrome (SARS-CoV) and Middle East respiratory syndrome (MERS-CoV), are capable of causing intestinal infection, besides respiratory tract infection [25,26]. SARS-CoV was also isolated by culture from intestinal biopsy specimens, and viral RNA could be detected in the stool of patients for more than 10 weeks after symptom onset [25]. MERS-CoV can be released in polarized intestinal epithelial cells after infection. The human gastrointestinal tract can serve as an alternative route to acquire the MERS-CoV infection [26]. Considering the similarity of biological characteristics of SARS-CoV-2 with SARS-CoV and MERS-CoV, and the fact that gastrointestinal involvement of SARS-CoV-2 infection and the isolation of live virus from the stool samples of the patients with COVID-19, it was believed that SARS-CoV-2 could be transmitted by the digestive tract via “fecal-oral” and “fecal-respiratory” mode. A study on a family-clustered infection with SARS-CoV-2 showed that two young adults from the family had diarrhea, suggesting that the gastrointestinal transmission route may play an important role in disease transmission [27]. However, until now, experiments fulfilling Koch’s postulates by using virus isolated from the feces of COVID-19 patients have not yet been reported. Therefore, there is no definite conclusion about the fecal-oral transmission of COVID-19. Although circumstantial evidence suggests that SARS-CoV-2 may be transmitted through feces, more efforts are needed to determine the role of fecal-oral transmission.

SARS-CoV-2 is a brand-new virus, and its routes of transmission and modes of transmisson still not fully understood. It is necessary to prevent the virus from entering the possible transmission routes in advance. The preventive measures should be increased so as to block the virus transmission through the digestive tract, which is also conducive to epidemic prevention and control. Thus, clinicians, especially gastroenterologists, should pay close attention to atypical symptoms of the digestive system of patients with COVID-19. The medical staff should ensure personal protection from the vomitus and feces of these patients. The excrement of patients and suspicious patients in hospital should be discharged after being disinfected. The virus in feces can easily enter the human food link through sewage; therefore, attention should be paid to the fecal sludge management and food safety.
